# Patterns of response of breast cancer after neoadjuvant chemotherapy according to molecular subtype

**DOI:** 10.3389/fonc.2025.1670594

**Published:** 2025-11-26

**Authors:** Malak Ghezzawi, Mohamad Hadi El Charif, Lynn Lteif, Vahe Panossian, Anthony Haddad, Sali Sarkis, Yara Zebian, Mira Kheil, Mohammad Ali Maktabi, Najla Fakhruddin, Jaber Al Abbas, Hazem Assi, Ziad Salem, Eman Sbaity

**Affiliations:** 1 Department of Surgery, American University of Beirut, Beirut, Lebanon; 2 Department of Internal Medicine, American University of Beirut, Beirut, Lebanon; 3 Faculty of Medicine, American University of Beirut, Beirut, Lebanon; 4 Department of Pathology and Laboratory Medicine, American University of Beirut, Beirut, Lebanon

**Keywords:** breast cancer, neoadjuvant chemotheraphy, pathological complete response, molecular subtype, residual disease

## Abstract

**Background:**

Neoadjuvant chemotherapy (NACT) is a key component in the treatment of locally advanced breast cancer, with pathologic complete response (pCR) varying by molecular subtype. This study evaluates pCR rates following NACT in a Lebanese cohort, stratified by molecular and clinicopathological features.

Methods: This retrospective cohort included 187 women with stage T1–T4, N1, M0 breast cancer treated at the American University of Beirut Medical Center between 2010 and 2016. Molecular subtypes (Luminal A, Luminal B, HER2-positive, triple-negative) were determined by immunohistochemistry and/or FISH. pCR was defined as ypT0N0. Univariate and multivariate analyses were performed to identify predictors of pCR.

Results: The median age was 45 years. Molecular subtype distribution was: Luminal A (28.9%), Luminal B (45.5%), HER2-positive (11.8%), and triple-negative (13.9%). Overall pCR was achieved in 12.8% (24/187) of patients. pCR rates significantly differed across molecular subtypes: HER2-enriched 45.5%, Luminal B 11.8%, triple-negative 11.5%, and Luminal A 1.9% (p = 0.005). Axillary involvement was also significantly associated with pCR (p < 0.001), while clinical T stage, tumor grade, focality/centricity, and chemotherapy regimen showed no significant associations. In multivariable logistic regression, molecular subtype remained an independent predictor of pCR (p = 0.002).

**Conclusion:**

pCR rates varied markedly among molecular subtypes, being highest in HER2-enriched tumors and lowest in Luminal A. These findings support molecular subtype as a key determinant of treatment response and highlight its role in guiding neoadjuvant treatment strategies.

## Introduction

Breast cancer is one of the most diagnosed cancers globally, according to GLOBOCAN 2022 2.3 million cases are reported (1). In the United States, 310, 720 are diagnosed with invasive breast cancer in 2024, and approximately 42, 250 die from their disease (2). The disease continues to be a leading cause of death among women (3–5). In Lebanon, breast cancer incidence and mortality rates are among the highest globally (6, 7).

Breast cancer therapeutic modalities include surgical resection, chemotherapy, radiotherapy, and hormonal therapy (8, 9). Currently, neoadjuvant chemotherapy (NACT) is established as the primary approach for the treatment of locally advanced breast cancer (10). This strategy proved its effectiveness in presurgical tumor size reduction, thus enhancing the chances of a successful breast-conserving surgery (BCS) and improving the overall treatment outcomes (11). Additionally, NACT serves evaluation of the tumor’s response to chemotherapy *in vivo* and it targets and treats any existing micrometastases (11). NACT gained prominence over the past two decades, showing significant benefits in long-term survival, disease-free survival, and progression-free survival (12–17). NACT currently, it is a fundamental component in the comprehensive management of breast cancer (10, 18).

The response to NACT can vary significantly depending on the molecular subtype of breast cancer (19–22). Triple-negative and HER2+ subtypes often show higher rates of pathologic complete response (pCR), with approximately 30% to 40% of patients achieving pCR (19, 20). In contrast, luminal A and luminal B subtypes generally have lower pCR rates, ranging from 10% to 25% (20–22). Achieving pCR is associated with improved overall survival and lower recurrence rates (14, 17). However, partial response (PR), defined as ≥50% reduction in tumor size, also plays a significant role (23). A meta-analysis conducted in 2018 by the Early Breast Cancer Trialists’.

Collaborative Group, involving 4, 756 women with early breast cancer, revealed that more than two-thirds (69%) of patients undergoing NACT achieved either a partial or complete clinical response (23). The extent of this response influenced surgical decisions, with a higher proportion of those achieving pCR opting for BCS (83% of 544) compared to those with PR (68% of 799) or no response (NR) (42% of 588) (23). Despite these differences in surgical choices, women achieving a pCR did not have a significantly lower rate of local recurrence compared to those with PR or NR (23). Nonetheless, both pCR and PR were associated with reduced rates of distant recurrence and mortality compared to no response (23). Understanding the differences in pCR and PR rates across various subtypes is crucial for optimizing treatment strategies and tailoring approaches to individual patient needs (22, 23). For instance, while triple-negative patients gain considerable benefits from achieving pCR, HER2+ patients might exhibit varying outcomes with partial responses (22), highlighting the necessity for personalized treatment strategies.

Given the increasing incidence of breast cancer in Lebanon and the proven effectiveness of NACT, it is crucial to identify specific prognostic factors associated with this treatment. This study aims to contribute to the existing literature by describing the pathological response obtained with NACT according to the molecular classification of breast cancer in Lebanese patients, offering valuable insights for future treatment approaches.

## Methods

### Study population

This study is approved by the Institutional Review Board at the American University of Beirut Medical Center (AUBMC) under protocol number BIO-2020-0013. Given the retrospective nature of the study, informed consent is waived.

A retrospective chart review is conducted of patients presented to AUBMC between January 2010 and December 2016. The patients’ data is extracted from a breast cancer database developed from the Medical Archives Department. The study focuses on Lebanese women aged 18 years and older, initially diagnosed with T1–4 N1 M0 breast cancer, and received neoadjuvant chemotherapy. Patients who underwent total mastectomy with sentinel lymph node biopsy and/or axillary lymph node dissection, or subsequent BCS were included, while patients who did not receive any breast cancer treatment at AUBMC were excluded.

### Data collection

Data of included patients is collected from the medical records under the following headings: demographics (age and gender), medical and family history (comorbidities, breast cancer, and other cancer types), clinical presentation (palpable mass and nodes), initial evaluation of the breast tumor (ultrasound, mammography, MRI, PET-CT, fine needle aspiration, and biopsy), tumor characteristics (biopsy pathology and immunohistochemistry), neoadjuvant chemotherapy (NACT) regimen, tumor evaluation after NACT (ultrasound, mammography, MRI), response to chemotherapy of the primary tumor and sentinel lymph nodes (complete, partial, or no response), type of breast surgery (total or partial mastectomy), axillary surgery (sentinel lymph node biopsy, axillary lymph node dissection), surgical findings (surgical pathology), and pathologic staging.

### Classification of molecular subtypes

Molecular subtypes are classified based on immunohistochemistry and/or fluorescence *in situ* hybridization (FISH) assays for estrogen receptor (ER), progesterone receptor (PR), HER2 and the proliferation marker Ki-67. The study population is categorized into four molecular subtypes according to standard definitions (24–26). Luminal A tumors were defined as ER and/or PR positive, HER2 negative, and Ki-67 <20%. Luminal B included two categories: (1) Luminal B (HER2 -negative), defined as ER and/or PR positive, HER2 negative, and Ki-67 ≥20%; and (2) Luminal B (HER2-positive), defined as ER and/or PR positive and HER2 positive, irrespective of Ki-67 level. The HER2-enriched subtype was defined as ER and PR negative with HER2 positivity. Finally, triple-negative breast cancer was defined as ER, PR, and HER2 negative. The Ki-67 cutoff of 20% was consistently applied to distinguish Luminal A from Luminal B (HER2-negative) tumors, in line with international consensus guidelines, including the 13th St. Gallen International Breast Cancer Conference (26).

### Study endpoints

Clinical response is defined as a reduction in T and/or N stages, while no response is defined as an increase in T and/or N stages or no change. pCR is defined according to Chevallier’s criteria (27), which characterize pCR as the absence of both invasive and *in situ* carcinoma in the breast and axillary nodes (ypT0N0). The primary endpoint is the rate of pCR according to molecular subtypes. Secondary endpoints include pCR rates in relation to clinical variables such as age (stratified as ≤40 years or >40 years), clinical T stage, clinical N stage, focality, histologic type, grade, NACT regimen, and number of NACT cycles (≤4 or >4). Additionally, patient and tumor characteristics are compared between those who achieve pCR and those who do not.

### Statistical analysis

Statistical analyses were performed using IBM SPSS version 29.0 (IBM, Chicago, USA). Descriptive statistics were conducted for all variables. Pearson’s Chi-square test was used to compare patients who achieved pCR with those who did not, with statistical significance set at p < 0.05.

Variables with clinical relevance or p < 0.1 in univariate analysis were entered into a multivariable logistic regression model to identify independent predictors of pCR. Molecular subtype was included as a categorical variable, with Luminal A serving as the reference group; therefore, adjusted odds ratios (aORs) represent comparisons relative to Luminal A. Other variables included in the model were age, clinical T stage, clinical N stage, focality, histologic type, tumor grade, NACT regimen, and number of NACT cycles. Adjusted odds ratios (aORs) with 95% confidence intervals (CIs) were reported.

## Results

### Patient characteristics

A total of 187 patients are included. **Table 1** presents the characteristics and demographics of these patients. The median age of is 45 years, with a range of 25 to 84 years. 167 (89.3%) patients have invasive ductal carcinoma (IDC), 14 (7.5%) have invasive lobular carcinoma (ILC), and 1 (0.5%) has both IDC and ILC. Before NACT, the Tumor classifications demonstrated T1 (27.8%), T2 (60.5%), and T3 (11.8%), axillary node involvement was N0 observed in 58 (31.2%) and N1-N3, in 128 (68.8%) patients. Tumor grade distribution is 23 (12.3%) for grade 1, 73 (39.0%) for grade 2, and 90 (48.1%) for grade 3. 111 (59.4%) patients have unifocal breast cancer, 51 (27.3%) have multifocal tumors, and 25 (13.4%) have multicentric tumors.

**Table 1:** Patient characteristics.

Characteristic	Number (n=187)	Percentage
Age		
Median (range)	45 (25-84)	–
T-stage prior to NACT		
T1	52	27.8
T2	113	60.4
T3	22	11.8
Axillary involvement		
N0	58	31.2
N1-N3	128	68.8
Tumor histology		
Invasive lobular carcinoma (ILC)	14	7.5
Invasive ductal carcinoma (IDC)	167	89.3
ILC and IDC	1	0.5
Others	5	2.7
Tumor grade		
1	23	12.3
2	73	39.0
3	90	48.1
Unknown	1	0.5
Focality and centricity		
Unifocal	111	59.4
Multifocal	51	27.3
Multicentric	25	13.4
Molecular subtype		
Luminal A	54	28.9
Luminal B	85	45.5
HER2	22	11.8
Triple negative	26	13.9

### Distribution of molecular subtypes

In this study, 28.9% (54 patients) are Luminal A tumors, 45.5% (85 patients) are Luminal B, 11.8% of cases (22 patients) are HER2-positive tumors and 13.9% (26 patients) are triple- negative tumors (**Table 1**).

### Treatment regimens and imaging modalities

104 patients (55.6%) undergone total mastectomy, and 83 patients (44.4%) have undergone partial mastectomy. 116 patients (62.0%) had axillary lymph node dissection and 71 patients (38.0%) had sentinel lymph node biopsy. Among these patients, 112 (59.9%) receive AC-T, 42 (22.5%) receive FEC or FEC-T, and 31 (16.6%) receive a combination of therapies or other treatments. Additionally, 2 patients (1.1%) have missing data regarding their treatment regimen. The median number of NACT cycles is 12, with 23.5% of patients receiving 6 cycles or fewer and 74.9% receiving 7 cycles or more. Trastuzumab (Herceptin) was administered to 60/187 patients (32.1%). Among HER2-positive patients, 96.7% received trastuzumab, reflecting high adherence to targeted therapy protocols in our cohort (**Table 2**). The imaging modalities post NACT were mammogram for 33.7% of patients (63 patients), ultrasound for 39.6% of patients (74 patients), and MRI for 16.6% of patients (31 patients) (**Table 2**).

**Table 2:** Treatments administered to cohort patients.

Characteristic	Number (n=187)	Percentage
Breast surgery to treatment		
Total mastectomy	104	55.6
Partial mastectomy	83	44.4
Axilla surgery		
Axillary lymph node dissection	116	62.0
Sentinel lymph node biopsy	71	38.0
Neoadjuvant chemotherapy regimen		
AC-T	112	59.9
FEC and FEC-T	42	22.5
Combination/Others	31	16.6
Missing	2	1.1
Number of NACT cycles		
Median (range)	12 (4-17)	
6 or less	44	23.5
7 or more	140	74.9
Use of herceptin		
No	127	67.9
Yes	60	32.1
Use of herceptin, in case of HER2 positive cases		
No	2	3.3
Yes	58	96.7
Imaging modality Re-evaluating the Cancer after NACT		
Mammogram		
No	124	66.3
Yes	63	33.7
Ultrasound		
No	113	60.4
Yes	74	39.6
MRI		
No	156	83.4
Yes	31	16.6

### Response in primary tumor and axilla according to breast cancer subtypes

**Table 3** presents the degree of pathologic response in the primary tumor and axilla according to molecular subtype. In the primary tumor, HER2-positive tumors exhibit the highest complete response (CR) rate at 63.6% (n = 14/22), followed by triple-negative tumors at 38.5% (n = 10/26), Luminal B tumors at 25.9% (n = 22/85), and Luminal A tumors at 9.3% (n = 5/54). For partial response (PR), the highest rate is observed in Luminal B tumors at 60.0% (n = 51/85), followed by Luminal A at 64.8% (n = 35/54), triple-negative at 46.2% (n = 12/26), and HER2- positive tumors at 27.3% (n = 6/22). These differences in primary tumor response are statistically significant (p < 0.01).

**Table 3:** Degree of pathologic response in the primary tumor and axilla reported separately according to molecular subtype.

Subtype	Total	No response		Partial response		Complete response*		p- value
		Number	Percentage	Number	Percentage	Number	Percentage	
Primary tumor								
Luminal A	54	14	25.9	35	64.8	5	9.3	<0.01
Luminal B	85	12	14.1	51	60.0	22	25.9	
HER2	22	2	9.1	6	27.3	14	63.6	
Triple negative	26	4	15.4	12	46.2	10	38.5	
Total	187	32	–	104	–	51	–	
Axilla (N1-N3)								
Luminal A	32	18	56.3	10	31.3	4	12.5	<0.01
Luminal B	63	23	36.5	17	27.0	23	36.5	
HER2	16	3	18.8	0	0	13	81.3	
Triple negative	17	6	35.3	4	23.5	7	41.2	
Total	128	50	–	31	–	47	–	

* Complete response refers to absence of tumor in the breast or axilla individually.

In the axilla, HER2-positive tumors again show the highest complete response rate at 81.3% (n = 13/16), followed by triple-negative tumors at 41.2% (n = 7/17), Luminal B tumors at 36.5% (n = 23/63), and Luminal A tumors at 12.5% (n = 4/32). For no response, Luminal A tumors have the highest rate at 56.3% (n = 18/32), followed by Luminal B at 36.5% (n = 23/63), triple- negative at 35.3% (n = 6/17), and HER2-positive tumors at 18.8% (n = 3/16). These differences in axillary response are also statistically significant (p < 0.01).

### pCR according to breast cancer subtypes

Out of the 187 patients, 24 achieved a pathologic complete response (pCR) following neoadjuvant chemotherapy (NACT), resulting in an overall pCR rate of 12.8%. **Table 4** presents analysis of pCR rates stratified by molecular subtype. Significant differences are observed across subtypes (p = 0.005). The HER2-positive subtype exhibits the highest pCR rate at 45.5% (n = 10/22), followed by Luminal B at 11.8% (n = 10/85), Triple Negative at 11.5% (n = 3/26), and Luminal A with the lowest rate at 1.9% (n = 1/54). Multiple logistic regression confirmed that molecular subtype was an independent predictor of pCR (p = 0.002) (**Table 5**).

**Table 4:** Pathologic complete response (pCR) according to molecular subtype and clinicopathological characteristics (univariate analysis).

Characteristics	Total	No pCR		pCR		p-value
		Number (n=163)	Percentage	Number (n=24)	Percentage	
Molecular subtype						
Luminal A	54	53	98.1	1	1.9	0.005
Luminal B	85	75	88.2	10	11.8	
HER2	22	12	54.5	10	45.5	
Triple negative	26	23	88.5	3	11.5	
Age*						
≤40	47	45	95.7	2	4.3	0.030
>40	140	118	84.3	22	15.7	
T-stage prior to NACT						
T1	52	43	82.7	9	17.3	0.260
T2	113	100	88.5	13	11.5	
T3	22	20	90.9	2	9.1	
Axillary involvement						
N0	58	58	100.0	0	0.0	<0.001
N1-N3	128	104	81.3	24	18.8	
Focality and centricity						
Unifocal	111	97	87.4	14	12.6	0.753
Multifocal	51	45	88.2	6	11.8	
Multicentric	25	21	84.0	4	16.0	
Grade						
1	23	21	91.3	2	8.7	0.091
2	73	67	91.8	6	8.2	
3	90	74	82.2	16	17.8	
Neoadjuvant chemotherapy regimen						
AC-T	112	100	89.3	12	10.7	0.473
FEC and FEC-T	42	34	81.0	8	19.0
Combination/Others	31	27	87.1	4	12.9
Missing	2				
Number of NACT cycles						
4 or less	5	3	60.0	2	40.0	0.128
5 or more	179	157	87.7	22	12.3	

*Although age remained in the analysis, no narrative interpretation was provided due to the cohort’s skewed age distribution (75% ≥40 years), to avoid overinterpretation.

**Table 5:** Multiple logistic regression analysis of predictive factors for pathologic complete response to neoadjuvant chemotherapy.

Variable	aOR (95% CI)	p-value
Age at Diagnosis*		***0.039***
≤ 40 years (reference)	1	
> 40 years	6.78 (1.10–41.81)	
Breast Cancer Molecular Subtype**		***0.002***
Luminal A (reference)	1	—
Luminal B	6.85 (0.71–65.76)	0.096
HER2-enriched	42.41 (3.94–457.02)	***0.002***
Triple-negative	8.38 (0.63–112.26)	0.109
Type of Breast Cancer		0.967
IDC (reference)	1	—
ILC	1.08 (0.11–10.25)	0.947
Mixed IDC + ILC	0.00 (0.00–.)	1.000
Tumor Grade		0.916
Grade 1 (reference)	1	—
Grade 2	0.49 (0.07–3.32)	0.466
Grade 3	0.91 (0.14–5.79)	0.919

Adjusted Odds ratios (aOR) are shown with corresponding 95% confidence intervals (CI) and p-values.

*Although age remained in the analysis, no narrative interpretation was provided due to the cohort’s skewed age distribution (75% ≥40 years), to avoid overinterpretation.

**Reference category Luminal A. Subtype entered as a four-level categorical variable. aORs are vs Luminal A. Bold values indicate statistical significance at p < 0.05.

### pCR rates according to other clinicopathological variables

The impact of various clinicopathological variables on pCR rates was assessed. These variables included age at diagnosis (<40 years vs. ≥40 years), clinical T stage, focality and centricity, tumor grade, axillary involvement, and neoadjuvant chemotherapy regimen. Univariate analysis showed that only axillary involvement (p < 0.001) was significantly associated with pCR (**Table 4**). All patients who achieved pCR were classified as N1–N3, with no pCR observed among patients with N0 disease (p < 0.001). Conversely, clinical T stage, focality and centricity, tumor grade, neoadjuvant chemotherapy regimen, and the number of NACT cycles did not show statistically significant associations with pCR (all p > 0.05) (**Table 4**).

Multiple logistic regression analysis confirmed that breast cancer molecular subtype (p = 0.002) was an independent predictor of pCR.

## Discussion

This study provides a comprehensive examination of the pathologic response to NACT across different molecular subtypes of breast cancer in Lebanon.

This cohort demonstrates a median age of our cohort aligns concordant with age range of 50–70 years reported in similar studies, reflecting a consistent demographic profile (19 -21, 28). The distribution of surgical procedures and tumor staging observed corresponds with established patterns in the literature (28, 29). Similarly, the higher proportion of T2 tumors is consistent with reported studies where approximately 60% of cases are T2 tumors (28, 29).

The distribution of breast cancer subtypes in this study matches the general patterns described in the literature. According to Perou’s classification, of all invasive cancers, Luminal A tumors account for 30%–40%, Luminal B HER2-negative tumors for 20%–30%, HER2-positive tumors for 12%–20%, and triple-negative tumors for 15%–20% (30). The distribution of breast cancer subtypes in our sample (Luminal A, Luminal B, HER2+, and triple-negative) mirrors findings from other studies, indicating a representative sample (28, 31).

This cohort had an anthracycline and taxane-based regimens, treatment pattern as a treatment with a pattern aligning with the established NACT protocols (29, 31). Treatment decisions are subtype-dependent, reflecting the personalized approach adopted in this study. Luminal B is the most prevalent subtype, highlighting the importance of downstaging tumors and increasing the likelihood of breast-conserving surgery (BCS), particularly in Luminal B and HER2+ subtypes (28, 31).

The pCR rate achieved in this study is 12.8%, lower than those reported in neighbouring regions and international studies. For instance, studies in Morocco (32) and Iraq (33) report pCR rates of 28% and 29.2%, respectively, which are significantly higher than our findings. The partial response rate observed in Iraq (70.8%) is also notably higher, suggesting regional differences in treatment response (33). These discrepancies could reflect variations in treatment protocols, patient compliance, or other factors that are not captured in our study, such as differences in the duration of treatment or follow-up. When compared with international studies, our pCR rate is also lower than that reported in studies by Diaz et al. (19), Cirier et al. (34), and Haque et al. (21), which report pCR rates ranging from 15.2% to 19%. In contrast, the findings of Cortazar et al., which show a pCR rate of 13.0%, align more closely with our results (31). However, a study by Pastorello et al. observes a much higher pCR rate of 26.6% (20). These variations highlight the substantial variability in pCR rates across different studies. It is essential to consider that differences in study designs, treatment protocols, and patient populations, such as compliance, tumor characteristics, and the type of neoadjuvant chemotherapy used, may account for these discrepancies. While our study’s findings are generally consistent with the broader trends, the variations in pCR rates emphasize the importance of regional and population-specific factors when interpreting treatment outcomes.

This study revealed that HER2-positive and Luminal B tumors have the highest pCR rates, while Luminal A tumors have the lowest pCR rate. These findings align with a Latin American study by Diaz-Casas et al., which showed that Luminal B tumors exhibited the highest pCR rate (54.5%), followed by triple-negative tumors (20.8%), HER2-positive tumors (16.9%), and Luminal A tumors (7.8%) (19). The differences in pCR rates between Luminal B and HER2 - positive tumors in our study are modest compared to the findings by Pastorello et al., where out of 177 patients who achieved pCR, 19 had HR+/HER2− tumors (10.7%), 80 had HER2+ tumors (45.2%), and 78 had triple-negative tumors (44.1%) (20). While the general trends are similar, it’s important to note that our study shows a notably higher pCR rate for Luminal B tumors compared to the other studies. A study by Haque et al., which included 13, 939 women, reported a pCR rate of 38.7% for HER2-positive cancers, with lower rates for triple- negative tumors (23.2%), Luminal B tumors (8.3%), and Luminal A tumors (0.3%) (21), which also reflects the trends observed in our study. Although the pCR rates for HER2 -positive tumors are comparable to those found in other studies, the higher pCR rate for Luminal B tumors in our study, alongside the lower pCR rates for Luminal A and triple-negative tumors, indicates some regional and study-specific variations. These differences underscore the importance of considering regional factors when interpreting pCR outcomes.

In our cohort, age was included in the analysis but not interpreted narratively due to the skewed age distribution, with more than 75% of patients aged ≥40 years. Previous studies, such as Chou et al., have reported higher pCR rates among younger patients (<50 years), particularly in HER2-positive and triple-negative subtypes (35). These differences may be attributed to variations in population demographics, treatment protocols, and sample size across studies. Because of the age imbalance in our cohort, any apparent association between age and pCR should be considered inconclusive. Larger, prospective studies with balanced age distribution are needed to better determine the role of age in predicting response to NACT.

Our study assesses the effect of NACT on pCR, a potential prognostic indicator for patients with breast cancer. This investigation is the first of its kind in Lebanon and is unique in encompassing all breast cancer subtypes. By contributing novel insights to the global literature and enriching regional evidence, our study aligns with comparable international research aimed at understanding the relationship between NACT and pCR in breast cancer patients (36, 37).

One of the main strengths of this study lies in the high quality of the information obtained. The data were collected through a rigorous process of reviewing medical records, ensuring both accuracy and reliability. The findings of this study provide valuable insights and inform the approach and treatment of patients with locally advanced breast cancer at AUB-MC and in Lebanon. The robustness of the data collection, verification, and analysis process ensures that the results can be relied upon to make informed decisions about patient care.

It is important to acknowledge several limitations of this study. First, the sample size was relatively small, which may limit the generalizability of the results. Second, the retrospective design carries an inherent risk of selection bias and incomplete clinical data. Third, the classification of Luminal B tumors remains heterogeneous in the literature, which may affect the precision of molecular subtype categorization. Additionally, the age distribution of our cohort was skewed toward patients aged ≥40 years; therefore, age was retained in the analysis but not interpreted narratively to avoid potential overestimation of its association with pCR. Furthermore, this study focused exclusively on patients treated with neoadjuvant chemotherapy and may not be generalizable to the broader breast cancer population. Lastly, long-term outcomes such as recurrence-free and overall survival were not assessed and should be addressed in future prospective studies.

## Conclusion

In conclusion, our study provides a comprehensive evaluation of the pCR to NACT across various breast cancer subtypes in Lebanon. The findings reveal a significant disparity in pCR rates, with HER2-positive and Luminal B subtypes demonstrating the highest response rates, while Luminal A tumors show the lowest. These results align with global trends but also highlight distinct regional variations, emphasizing the importance of incorporating local factors into treatment planning. This is the first study in Lebanon to encompass all major breast cancer subtypes, our research offers valuable insights into NACT effectiveness within the Lebanese population and enriches the regional evidence base. Despite limitations such as a relatively small sample size and the retrospective nature of the study, our findings advance the understanding of breast cancer treatment responses and underscore the necessity for personalized treatment strategies. Further research, particularly larger, prospective studies, is needed to validate these findings and explore long-term outcomes, ultimately improving breast cancer management and tailoring therapies to meet individual patient needs.

## Data Availability

The raw data supporting the conclusions of this article will be made available by the authors, without undue reservation.
